# Implementation of dispersion-free slow acoustic wave propagation and phase engineering with helical-structured metamaterials

**DOI:** 10.1038/ncomms11731

**Published:** 2016-05-20

**Authors:** Xuefeng Zhu, Kun Li, Peng Zhang, Jie Zhu, Jintao Zhang, Chao Tian, Shengchun Liu

**Affiliations:** 1 College of Physical Science and Technology, Heilongjiang University, Harbin 150080, China; 2 Department of Physics, Huazhong University of Science and Technology, Wuhan 430074, China; 3 Innovation Institute, Huazhong University of Science and Technology, Wuhan 430074, China; 4 State Key Laboratory of Transient Optics and Photonics, Chinese Academy of Sciences, Xi'an 710119, China; 5 Department of Mechanical Engineering, the Hong Kong Polytechnic University, Hung Hom, Kowloon, Hong Kong SAR, China; 6 Department of Biomedical Engineering, University of Michigan, Ann Arbor Michigan 48109, USA

## Abstract

The ability to slow down wave propagation in materials has attracted significant research interest. A successful solution will give rise to manageable enhanced wave–matter interaction, freewheeling phase engineering and spatial compression of wave signals. The existing methods are typically associated with constructing dispersive materials or structures with local resonators, thus resulting in unavoidable distortion of waveforms. Here we show that, with helical-structured acoustic metamaterials, it is now possible to implement dispersion-free sound deceleration. The helical-structured metamaterials present a non-dispersive high effective refractive index that is tunable through adjusting the helicity of structures, while the wavefront revolution plays a dominant role in reducing the group velocity. Finally, we numerically and experimentally demonstrate that the helical-structured metamaterials with designed inhomogeneous unit cells can turn a normally incident plane wave into a self-accelerating beam on the prescribed parabolic trajectory. The helical-structured metamaterials will have profound impact to applications in explorations of slow wave physics.

## Abstract (web summary)

There is great interest in slow wave propagation for a variety of applications. Here, Zhu *et al*. present a dispersion-free helical-structured metamaterial that implements acoustic wave deceleration at broad bandwidth and demonstrates specially designed phase modulation to incident sound through helicity tuning.

## Graphical abstract

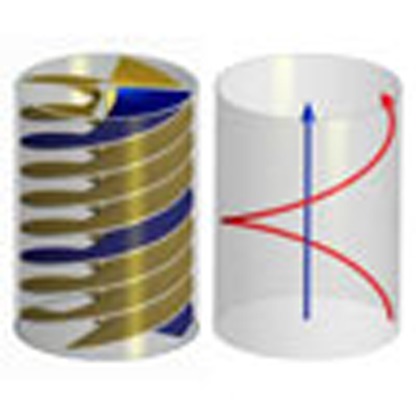

Marked control over the velocity at which wave propagates is a significant matter that remains unsolved, preventing from providing efficient ways to acquire many desired exotic functionalities in electronic, photonic, mechanical and acoustic systems. In the past decade, the phenomenon of slow wave propagation has been intensively explored in both optics^1^,^2^,^3^,^4^,^5^,^6^,^7^,^8^,^9^,^10^,^11^ and acoustics^12^,^13^,^14^,^15^,^16^,^17^,^18^, for the attempt to create advanced materials and devices that allow spatial compression of wave energy, wave signal buffering, and nonlinear effects enhancement. Typically, the proposed approaches^12^,^13^,^14^,^15^,^16^,^17^,^18^,^19^,^20^,^21^,^22^ can be categorized into two types. One takes advantage of the material resonances induced by waves. For example, waves can be slowed down inside dispersive materials near resonances. The other relies on resonances induced by the specifically designed structure. Both types of slow wave manipulations are resonance-based and therefore generally suffer from a very limited range of operating frequency^23^. To overcome the narrowband problem, techniques of adiabatic control over dispersion have been proposed to break the fundamental restriction on the attainable delay-bandwidth product, which eventually leads to the ‘rainbow' trapping effect^24^,^25^,^26^,^27^,^28^,^29^,^30^,^31^. Still, strong dispersion associated with rainbow trapping materials could cause massive distortion of pulse envelopes. As such, important applications including sequence signal buffering and wave pulse compression call for compact materials that support dispersion-free slow wave propagations.

In this paper, we present a type of dispersion-free helical-structured metamaterials that are able to slow down acoustic waves at broad bandwidth, by introducing helical wave rotation and wavefront revolution to the propagating waves. In our approach, the helical-structured metamaterials enabled sound deceleration can bring a notable phase change in the sub-wavelength scale. The phase change is decided by the helicity of the proposed metamaterials, hence tunable by adjusting the thread lead. Such flexibility is highly desirable in phase engineering applications^32^,^33^,^34^,^35^,^36^, such as designs of innovative ultrathin flat acoustic lenses, acoustic rectifiers, high efficient couplers for surface acoustic waves and self-accelerating beam generators^13^,^14^,^15^,^16^,^17^,^18^,^37^,^38^,^39^,^40^. Very different from the previously reported approaches^15^,^16^,^17^,^18^, our work uncovers the non-dispersive nature of helical-structured metamaterials. Each frequency component of incident waves will see its velocity manipulated to the same extent. In previously reported approaches, the labyrinthine metamaterials can be mapped into an effective high-indexed medium inserted into a rigid block. Even though the high-indexed medium is effectively non-dispersive, the existing rigid background in the labyrinthine metamaterial still leads to dispersive acoustic impedance. From the effective medium point of view, the new helical-structured metamaterial, as a whole, is equivalent to an effective high-indexed medium without introducing extra rigid medium, providing high space utilization in the folding process and non-dispersive acoustic impedance (Supplementary Figs 1 and 2). The high space utilization is especially important for generating high-quality self-accelerating beams, where the output phase distribution of acoustic lens is rapidly modulated. Furthermore, given the same effective refractive index or space folding ratio, the effective mass density of air in the helical-structured metamaterial is much larger than the one in the labyrinthine metamaterial^15^,^16^,^17^,^18^, therefore, provides a highly competitive mass module candidate for the more complex spring-mass modelled resonance-based metamaterial.

## Results

### Helical-structured acoustic metamaterials

**Figure 1:**
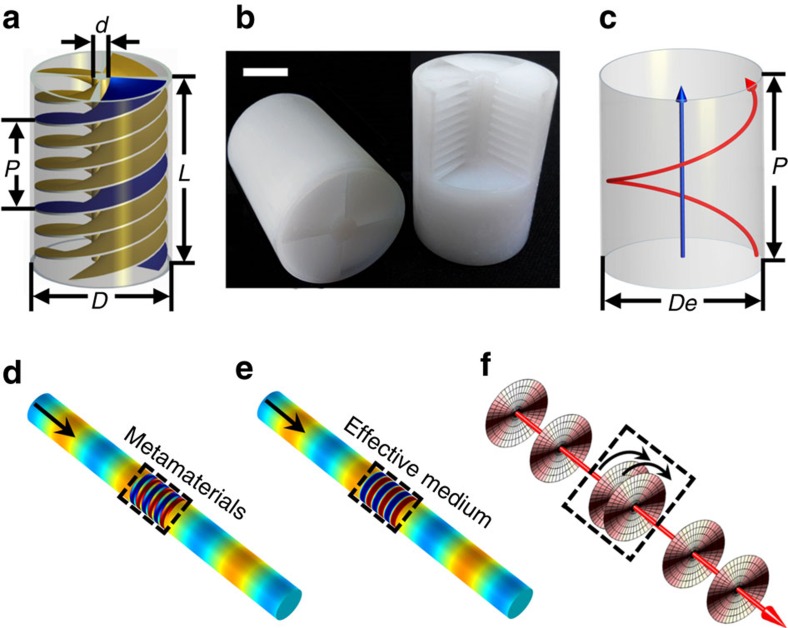
Helical-structured acoustic metamaterials. (**a**) A cylindrical helical-structured unit cell consists of four equally spaced wide blades spiralling around a slender shaft, the geometry of which is determined by outer diameter *D*, inner diameter *d*, overall length *L* and lead *P*. (**b**) Photograph of the fabricated helical-structured metamaterial unit cell samples with *D*=28 mm, *d*=6 mm, *L*=41 mm, and *P*=9 mm. Scale bar, 1 cm. (**c**) The acoustic wave travels along a helical path inside the structured metamaterials unit cell (the red arrow), while it can be treated as going straightly through an effective medium (the blue arrow). (**d**) Simulation result of sound pressure field distribution. The normally incident plane waves at 4,470 Hz propagate through the designed metamaterials unit cell in **b**. (**e**) Simulation result of sound pressure field distribution when the same incident waves in (**d**) propagate through an effective medium at *n*_eff_=5.6 and *ρ*_eff_=51.67 kg m^−3^, calculated from Equation (3). (**f**) The space between neighbouring acoustic wavefronts at the crest is shortened inside the metamaterials due to the helical propagation path around the central axis. Here the disks represent the acoustic wavefronts at the crest.

As shown in Fig. 1a,b, the helical-structured acoustic metamaterial consists of four spiralling blades spaced at 90° to each other, connected through a central slender column. Its geometric properties can be described by an outer diameter *D*, an inner diameter *d* much smaller than outer diameter, an overall length *L*, and a thread lead *P*, which is the distance along the unit cell's axis that is covered by one complete rotation of each blade (360°). All four geometric properties are smaller than the wavelength of sound in air *λ*_0_. Inside the metamaterials, acoustic waves are forced to propagate along a helical path instead of the normal straight route. As shown in Fig. 1c, assuming that the equivalent diameter of the helical path of sound is *D*_e_ and the thread lead *P* is much smaller than *λ*_0_, the refractive index *n* of the helical-structured metamaterials can be decided from the geometry to be the ratio between the helical path length and its projection on the propagation direction (Supplementary Fig. 3 and Note 1):


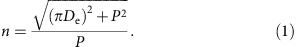


From the effective medium point of view, the helical-structured metamaterial behaves effectively as a homogeneous cylindrical metafluid column, with *n*_eff_ and *ρ*_eff_ being the effective refractive index and dynamic mass density along the axis, respectively. It is well-known that, the transmission coefficient of acoustic wave through a homogeneous cylindrical metafluid column of length *L* can be expressed as^41^:


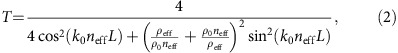


where **k**_0_ is the wave vector of sound in air and *ρ*_0_ is the mass density of air. It is worth noting that the effective refractive index and dynamic mass density can be retrieved from *T*, if taking advantage of the known transmission peaks associated with the Fabry–Pérot resonance modes. According to Equation (2), we can subsequently retrieve the expressions of *n*_eff_ and *ρ*_eff_ as follows (Supplementary Note 1):


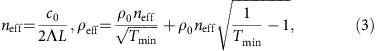


where *Λ* is the value of frequency where the fundamental Fabry–Pérot mode happens, *c*_0_ is the speed of sound in air, and *T*_min_ is the minimum transmittance in the transmission spectrum.

To indicate the results, we have performed full-wave simulations with both the helical-structured metamaterial and a homogeneous cylindrical metafluid column. For the metamaterial, the geometric properties are *D*=28 mm, *d*=6 mm, *L*=41 mm and *P*=9 mm. The effective refractive index and dynamic mass density of the metafluid column are set at *n*_eff_=5.6 and *ρ*_eff_=51.67 kg m^−3^, respectively, calculated from Equation (3), with the refractive index, mass density and speed of sound in air at room temperature being *n*_0_=1, *ρ*_0_=1.2 kg m^−3^ and *c*_0_=343.2 m s^−1^. The simulated pressure field distributions of acoustic wave traveling through the two materials are presented in Fig. 1d,e. Both pressure field distribution maps are closely identical, unequivocally revealing that the helical-structured metamaterial can be regarded as a homogeneous cylindrical metafluid column with high refractive index and large mass density defined by Equation (3). Intuitively, the acoustic wave is carrying a large wave vector k_eff_ or a shorten wavelength (*λ*_eff_=2*π*/k_eff_) in the metamaterial. Therefore, a strong local effect of spatial wave energy compression or concentration could be expected. Figure 1f conceptually describes such effect. The space between neighbouring wavefronts at crests is shortened inside the metamaterials due to the helical propagation path around the central axis. This leads to an enhanced wave–matter interaction, which imprints an auxiliary radiation mass to the vibrating air load as a form of dynamical back-action results. For the propagating acoustic waves, the air load inside the helical-structured metamaterials seems to be much heavier than that in free-space, resulting into a large effective dynamic mass density observed.

### Acoustic properties

**Figure 2:**
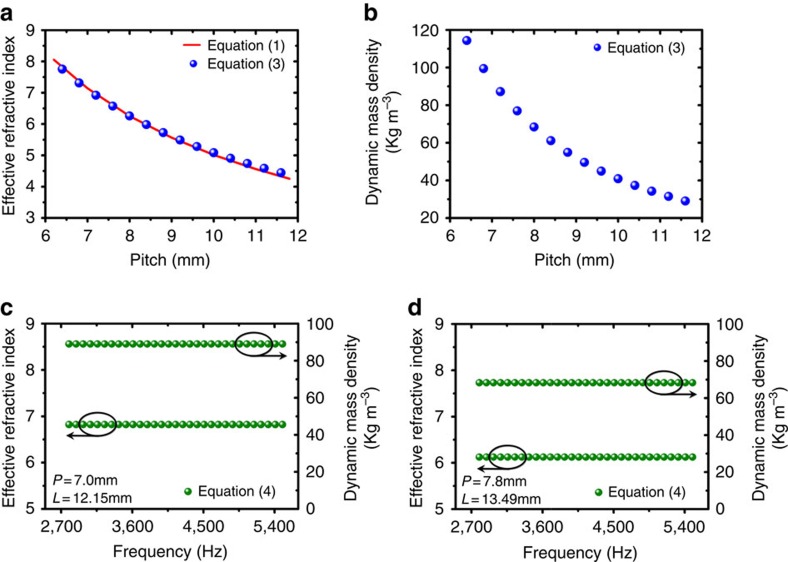
Effective material properties of the helical-structured acoustic metamaterials. (**a**) The refractive index *n* directly got from geometric parameters are calculated by Equation (1) and shown with the red curve versus lead *P*. The effective refractive index *n*_eff_ acquired using the effect medium approach are calculated by Equation (3) and shown with the blue sphere dots versus lead *P*. (**b**) The dynamic mass density *ρ*_eff_ acquired using effect medium approach are calculated by Equation (3) and shown with the blue sphere dots versus lead *P*. (**c**) The effective refractive indices *n*_eff_ and dynamic mass densities *ρ*_eff_ change with the frequency, calculated by Equation (4). Two different samples were studied. The first one has *L*=12.15 mm, *P*=7 mm. (**d**) The second sample has *L*=13.49 mm, *P*=7.8 mm. The flat lines demonstrate the non-dispersive nature of both samples. Notice that the arrows in **c** and **d** indicate the correspondences between the data lines and vertical coordinates.

For the helical-structured acoustic metamaterials, the effective parameters along the propagation direction are helicity dependent, subject to the variation by adjusting thread lead *P* when other geometric parameters are set. The changes of effective refractive index and dynamic mass density with thread lead *P* are demonstrated in Fig. 2a,b, respectively. Both factors are approximately in inverse relation with thread lead *P*. At about *P*=6.4 mm, the effective refractive index can reach high close to 8, while the inertia *ρ*_eff_ manifests a remarkable value with up to 120 kg m^−3^, two order of magnitude larger than that of air. It is worth mentioning here that the dynamic mass density of our designed metamaterials *ρ*_eff_≈2*ρ*_0_*n*_eff_/*T*_min_ is much larger than that (*ρ*_0_*n*_eff_) of labyrinthine metamaterials^15^,^16^,^17^,^18^, where the effective parameter *ρ*_0_*n*_eff_ can be simply derived from transformation acoustics by folding the zigzag channels into straighten ones.

Although the effective refractive index *n*_eff_ and dynamic mass density *ρ*_eff_ of the helical-structured metamaterials can shoot high with large helicity, they are non-dispersive in nature. That is, when the helicity of metamaterials is set, *n*_eff_ and *ρ*_eff_ remain the same for different frequency components. To verify its validity, we adopt the mathematics model used in developing the two-load four-microphone method, well-known for measuring complex transmission coefficients in an acoustic impedance tube (Supplementary Figs 4 to 5 and Note 2). From the 2 × 2 transfer matrix of a homogeneous and isotropic acoustic material with a finite thickness, we obtain that the relation between effective parameters and various spectral components can be calculated as follows:


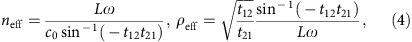


where *t*_*ij*_ are components of the transfer matrix for the metamaterial layer and *ω* is the angular frequency of acoustic waves in air (Supplementary Note 3). We consequently designed two helical-structured metamaterial samples with different helicities for calculations. The two metamaterials share the same outer diameter *D*, but one has the length *L*=12.15 mm, thread lead *P*=7 mm and the other has *L*=13.49 mm, *P*=7.8 mm. From the results depicted in Fig. 2c,d, the values of *n*_eff_ and *ρ*_eff_ are different for the two metamaterials due to their own separate helicities. However, it is evidently clear that for either metamaterials, the effective properties *n*_eff_ and *ρ*_eff_ remain unchanged within the studied frequency range. Unlike the strongly dispersive resonance-based slow sound materials, the flat spectra demonstrated by the helical-structured metamaterials suggest that such type of metamaterials is non-dispersive in nature, thus has the great capability to provide stable refractive index and mass density over a wide frequency range. It is worth to be pointed out that when the wavelength is decreasing downto the scale comparable to the sample size (∼20 mm), the metamaterial mode for the helical structure starts to break down. Both *n*_eff_ and *ρ*_eff_ will increase slightly at high frequency, which means it is no longer dispersion free (Supplementary Fig. 6).

### Experimental investigation

**Figure 3:**
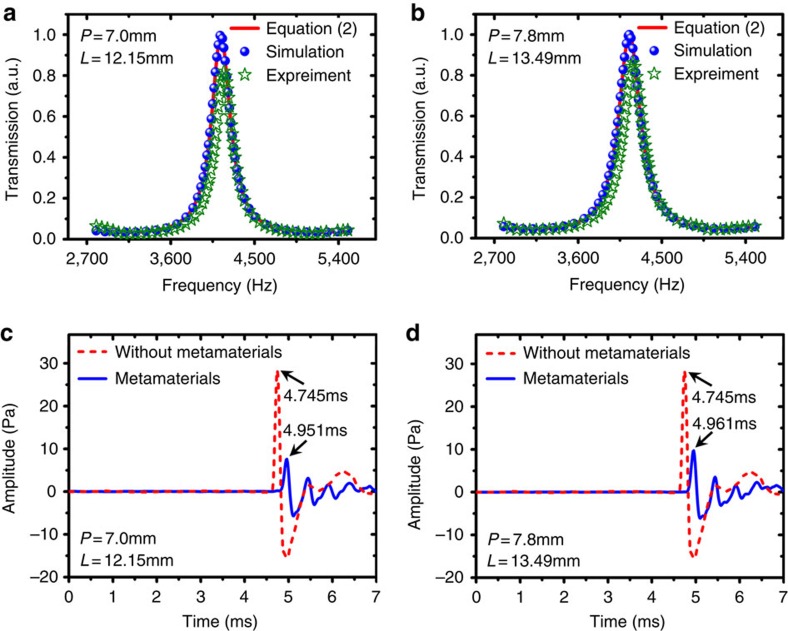
Demonstration of the acoustic properties of the helical-structured metamaterials. (**a**) Analytically, numerically calculated and experimentally measured transmission spectra for helical-structured metamaterial unit cell with *L*=12.15 mm, *P*=7 mm. (**b**) Analytically, numerically calculated and experimentally measured transmission spectra for helical-structured metamaterial unit cell with *L*=13.49 mm, *P*=7.8 mm. Both transmission peaks are located at around 4170 Hz. However, the transmitted components from the two samples possess different phase delays due to the different helical characteristics. (**c**) Time domain waveforms experimentally measured by the microphone. Transmitted signal passing through sample 1 in **a** is shown with blue solid curves, while the transmitted waveform through air is shown in red dashed curves. (**d**) Experimentally measured transmitted signal when replacing sample 1 with sample 2 in **b**.

To experimentally investigate the helical-structured acoustic metamaterials, we conducted through-transmission measurements on two actual samples whose geometric designs are the same as the ones involved in dispersion study and presented in Fig. 2. The samples are fabricated by using laser sintering stereo-lithography, a layer-by-layer additive manufacturing process capable of fabricating complex three-dimensional structures. The base material is photopolymer (Somos GP Plus) which is rigid enough with respect to air (Supplementary Fig. 7). The transmission performance of acoustic waves through the two samples are measured with a lab-made standing wave tube system (Supplementary Figs 4 to 5 and Note 2). We made sure that the largest thread lead of the metamaterial samples is smaller than 1/7 of the smallest incident wavelength used in our experimental investigation, so that the comparison between experimental results and analytical effective medium approach is meaningful. From the measured transmission spectra on two samples displayed in Fig. 3a,b, the transmission coefficients actually show smooth fluctuation with the frequency change, reaching sharp peaks at around 4,170 Hz. Both experimentally measured profiles are in good agreement with theoretical predictions produced through Equation (2) as well as numerical results from the full-wave simulations. The amplitudes of transmission peaks are measured to be less than unity due to the damping effect. Physically, the transmission peaks are attributed to the existence of Fabry-Pérot resonance modes inside the high-indexed metamaterial layers. It needs to be mentioned that the Fabry–Pérot resonance imposes a substantial restriction on high transmission in broadband. To overcome such a problem, we can employ the helical-structured metamaterial with progressive lead, which has much less acoustic impedance mismatch with air than the one with constant lead.

To further demonstrate the slow acoustic wave propagation caused by the helical-structured metamaterials, we sent out stable sound pulses with the central frequency 4,170 Hz and recorded transmitted time domain signals through no metamaterials and the two metamaterial samples in Fig. 3c,d. It is shown that the time interval for the pulses traversing from speaker to microphone without going through the metamaterials is around 4.745 ms. However, when the two metamaterial samples are separately placed in the sound propagation path, the received pulses exhibit 0.206 and 0.216 ms delay, which leads to effective metamaterial sound speeds of 50.3 and 52.8 m s^−1^, corresponding to over 80% drop from the normal sound speed in air. In Fig. 3c,d, the ringing pulses of transmitted signals passing through the metamaterial samples are mainly caused by multiple reflections at two high-reflective facets, which may be suppressed by introducing progressive lead. Here we would like to point out that such remarkable sound deceleration and signal delay are realized with the metamaterial samples of only 13 mm in length, and if we proceed to increase the helicity of such metamaterials, it has the potential to go even further.

### Generating an acoustic self-accelerating beam

**Figure 4:**
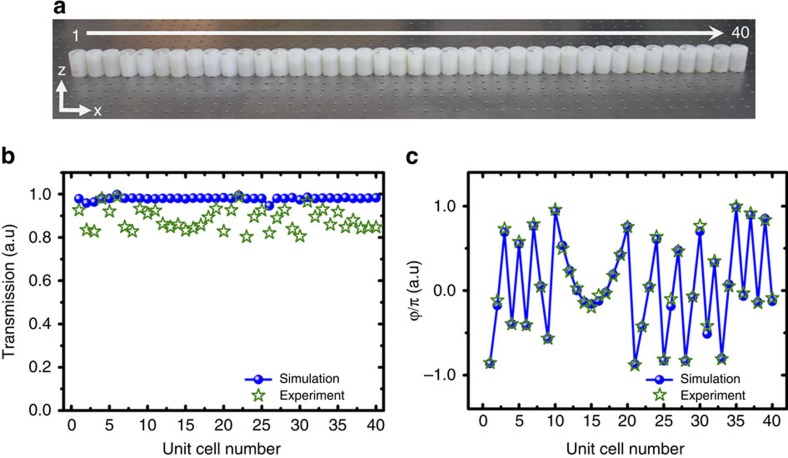
Meta-lens design for synthesizing an acoustic self-accelerating beam. (**a**) 40 helical-structured metamaterial unit cells were fabricated and utilized to construct acoustic meta-lens. (**b**) The transmission performance of each numbered meta-lens unit cell at 4,170 Hz. The experimental results are shown with green star dots, while blue sphere dots present numerical simulation outputs (**c**) The designed phase profile for synthesizing the acoustic self-accelerating beam. The blue dots represent the phase of each unit cell obtained from both the Equation (5) and the numerical simulation. The green star dots correspond to the experimentally measured phase modulation caused by each numbered unit cell. a.u., arbitrary unit.

The new helical-structured acoustic metamaterial is unique in a way that it has the capability to provide arbitrary phase delays to modulate the incident wave through helicity tuning. It can be utilized as a flexible building block to realize passive acoustic wave phase engineering. We consequently constructed a one-dimensional meta-lens to present an example of such possibility. As shown in Fig. 4a, the meta-lens is a one-dimensional anisotropic metamaterial that consists of an array of 40 helical-structured metamaterial unit cells. With spatially varying unit cell thread leads and lengths, the meta-lens can transform a normally incident plane wave into an acoustic self-accelerating beam along the prescribed parabolic route. To minimize the reflections, each unit cell is designed to operate nearby Fabry–Pérot resonances modes to maximize the overall transmission. According to the ray acoustics theory and Legendre transformations, the phase profile *ϕ*(*x*) at *z*=*z*_0_ for synthesizing an acoustic self-accelerating beam along the trace *f*(*z*) is determined by the equation^42^


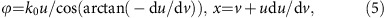


where


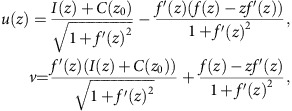



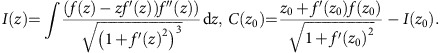


In our case, the position of the phase array is *z*_0_=−0.02 m and the predesigned acoustic self-accelerating beam curve is *x*=*f*(*z*)=−0.552(*z*−1.079)^2^+0.185. 40 helical-structured metamaterials unit cells were fabricated and separately measured. Each of the unit cell has its own helicity and length so that the phase delay it provides matches with the requirement calculated from Equation (5). From the measured transmission and phase delay of 40 unit cell samples plotted in Fig. 4b,c, the actual performance is in close agreement with the numerical simulation. Despite the inevitable thermo-viscous loss, overall throughput of each unit cells is tested to be over 80% at the frequency of interest with the loss factor *γ***<**0.003 (Supplementary Fig. 8).

**Figure 5:**
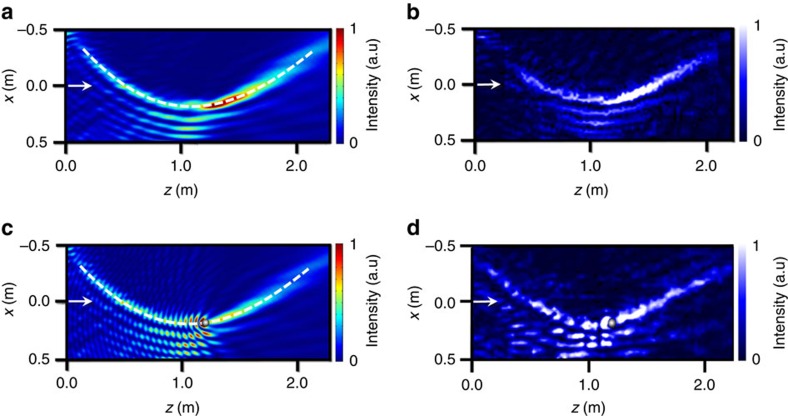
The acoustic self-accelerating beam generated by the meta-lens and the self-healing property. (**a**) The simulated pressure field distribution of generated acoustic self-accelerating beam. The operating frequency is 4,170 Hz. The dashed line marks the curved trajectory. The result shows that the generation of acoustic self-accelerating beam is barely affected by the coupling between adjacent unit cells in the meta-lens (Supplementary Fig. 11). (**b**) Experimental measurements of the pressure field distribution. (**c**) The simulation demonstration of the self-healing properties of acoustic self-accelerating beam. Here, a rigid cylinder (diameter: 4 cm) is placed at (*z*, *x*)=(1.211, 0.175) (m) on the curved trajectory. (**d**) The experimental demonstration of the self-healing properties with the same-sized obstacle made of aluminium alloy.

We further assembled the meta-lens with 40 fabricated unit cells (Supplementary Fig. 9 and Table 1) and tested it with continuous normally incident plane waves. In the panels of Fig. 5, we show the simulation and experimental results of acoustic self-accelerating beam generation by the meta-lens. The simulated transmitted acoustic field in Fig. 5a shows that an acoustic self-accelerating beam is formed and follows the predesigned parabolic curve, mimicking the ballistic motion of a projectile under the action of gravity. The measured result displayed in Fig. 5b matches well with the simulation output, revealing the exact same pattern. Fig. 5c,d subsequently demonstrate the self-healing property of the non-diffracting self-accelerating acoustic wave packet. In terms of the caustic nature of the beam structure, the wave packet is perfectly restored after the rigid obstacle. Therefore we have demonstrated the capability of helical-structured metamaterials in phase engineering.

## Discussion

We have proposed and demonstrated a helical-structured acoustic metamaterial, which enables dispersion-free slow wave propagation with a compact structure. Such metamaterials with large mass density are very desirable in many useful applications, such as implementing deep sub-wavelength resonant unit cells^43^ and boosting the radiation efficiency of sound sources^44^.The helicity-dependent refractive index of the metamaterials also provide a new way to passively engineer the phase of acoustic waves that will benefit the applications such as acoustic imaging and communication^13^,^14^,^15^,^16^, acoustic cloaking^42^,^45^,^46^,^47^,^48^ and particle manipulation^49^ and so on. A one-dimensional meta-lens has been constructed to test the hypothesis. The function of meta-lens as a beam shaper to transform a normally incident plane wave into an acoustic self-accelerating beam has been presented.

Our work provides a fertile ground for acoustic wave manipulations (acoustic waves rerouting, imaging, and holograms and so on) and fundamental explorations of slow wave physics. Of interest will be the extension of our work into nonreciprocal acoustics regime by integrating nonlinearity and time-varying, which shed lights on the developments of novel harmonics manipulation modalities and functional materials with topologically protected features^50^,^51^.

## Methods

### Numerical simulations

Three-dimensional numerical simulations are carried out by the finite element solver in commercial software COMSOL MultiphysicsTM 4.3b using a high performance computing cluster, to emulate the experimental conditions of the standing wave tube and the planar waveguide measurement system. The geometrical model of the helical-structured metamaterial layer is built up in commercial software Pro/Engineer, and then loaded into the acoustic-solid interaction module for the full-wave simulations. Perfectly matched layers are imposed on the outer boundaries of simulation domains to prevent reflections.

### Sample fabrication and experimental setup

The designed helical-structured metamaterial units are made of photosensitive resin (Somos GP Plus), and are manufactured via laser sintering stereo-lithography (SLA300, 75 micron). The transmission spectra of the metamaterial unit cells are measured in a lab-made impedance tube (Supplementary Fig. 4). A loudspeaker driven by a multifunctional signal generator (SRS MODEL DS345) and a power amplifier is mounted at one close end of the impedance tube. We insert a microphone(Brüel and Kjær type-4958 ¼ inch) into the tube at four different locations to record the time domain signals of sound pressure, with the other end opening or sealed with a rubber plug. By following the well-known four-microphone method, we first obtain the complex signals of sound pressure at different locations by using software PULSE Reflex Core, and then calculate the transmission of the metamaterial unit cells for each operating frequency.

In the planar waveguide system for the acoustic meta-lens experiment, we have used two paralleled plates (dimension: 3 m × 3 m × 15 mm), for which the spacing is 28 mm and equals to the outer diameter *D* of the metamaterial unit cells in the meta-lens. Sound absorbing foams are fixed at the boundaries of the planar waveguide to minimize reflections and isolate ambient noises. To generate the incident plane waves required for the experiment, 60 loudspeakers (AS04004PR-R, diameter: 40 mm) are assembled into a liner array with an interval of 44 mm. All the speakers are synchronously driven by a multifunctional signal generator (SRS MODEL DS345) combined with a lab-made power amplifier to emit same sound signal. For the pressure field measurement, we use a microphone to extract local pressures at different positions in the *x*–*z* plane. By employing commercial software PULSE Labshop, the acoustic intensity field is plotted after the pressure magnitudes at different spatial positions in the scanned region (2.3 m × 1.0 m) are recorded (Supplementary Fig. 10).

### Data availability

The data that support the findings of this study are available from the corresponding author on request.

## Additional information

**How to cite this article**: Zhu, X. *et al*. Implementation of dispersion-free slow acoustic wave propagation and phase engineering with helical-structured metamaterials. *Nat. Commun.* 7:11731 doi: 10.1038/ncomms11731 (2016).

## Supplementary Material

Supplementary Information Supplementary Figures 1-11, Supplementary Table 1, Supplementary Notes 1-3 and Supplementary References.
