# Supplementary material for: Implementation of dispersion-free slow acoustic wave propagation and phase engineering with helical-structured metamaterials
Source: Nat Commun. 2016 May 20;7:11731. doi: 10.1038/ncomms11731 (PMC4876457; doi:10.1038/ncomms11731)
Supplement: Supplementary Information — Supplementary Figures 1-11, Supplementary Table 1, Supplementary Notes 1-3 and Supplementary References. [file ncomms11731-s1.pdf]

a

b

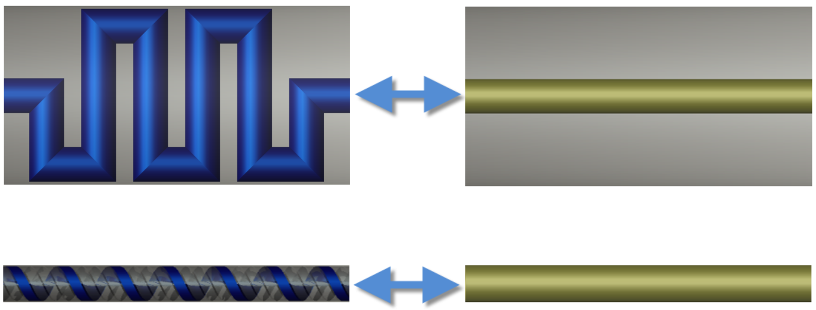

Supplementary Figure 1| The effective medium approaches. (a) Mapping the labyrinthine metamaterials into an effective high-indexed medium with an extra rigid background. **(b)** Mapping the helical-structured metamaterials into an effective high-indexed medium as a whole without introducing extra rigid parts. It is clearly shown that the designed structure in **(b)** provides a higher space utilization in the folding process.

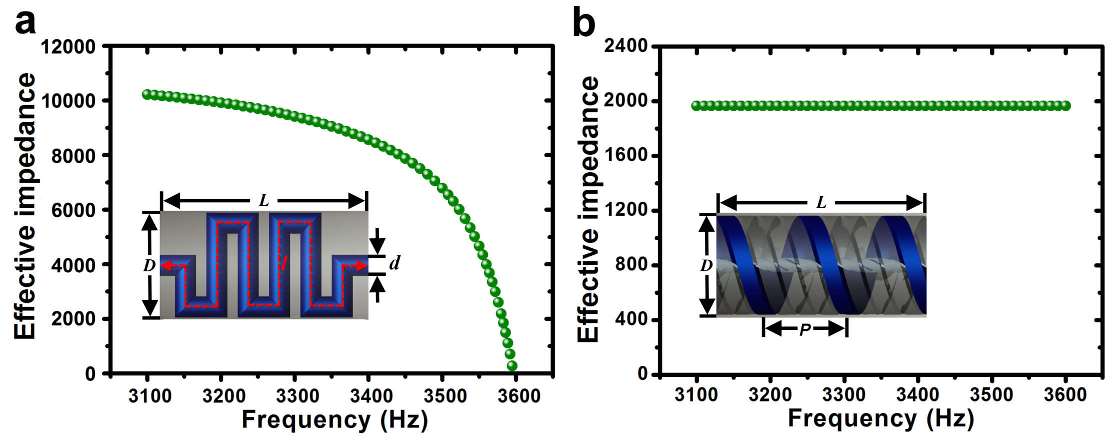

Supplementary Figure 2| The effective acoustic impedances (unit: kg·m^{2}·s^{-1}) between (a) the labyrinthine and (b) the helical-structured metamaterials. As shown in Supplementary Fig. 1, the labyrinthine metamaterials can be mapped into an effective high-indexed medium inserted into a rigid block. Even though the high-indexed medium is effectively non-dispersive, the existing rigid background in the labyrinthine metamaterial still leads to dispersive acoustic impedance (Supplementary Ref. 1), given by a numerical demonstration in **(a)**. The geometric parameters D=16 mm, L=28 mm, l=100 mm, and d=3 mm. In **(b)**, the helical-structured metamaterials, which can be mapped into an effective high-indexed medium as a whole, render non-dispersive acoustic impedance in the studied frequency range. The geometric parameters D=28 mm, L=22.51 mm, and P=13.4 mm.

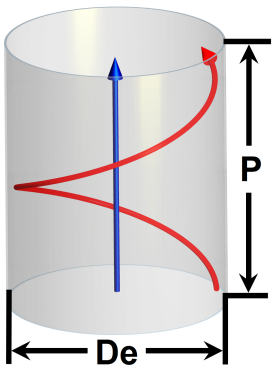

Supplementary Figure 3| Schematic diagram of the acoustic paths in the helical-structured metamaterials and the free-space. The acoustic path in the metamaterials is helical (the red arrow), while the path in free-space is straight (the blue arrow).

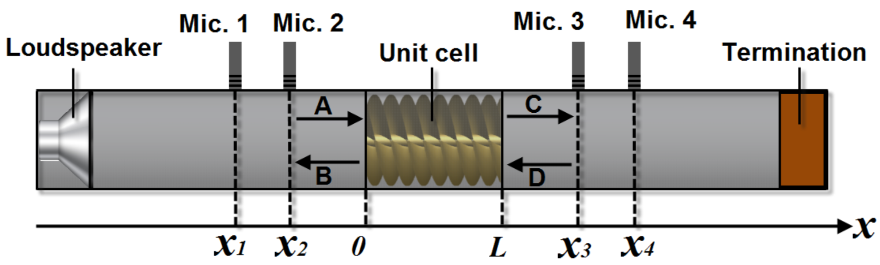

Supplementary Figure 4| Schematic of the lab-made acoustic impedance tube.

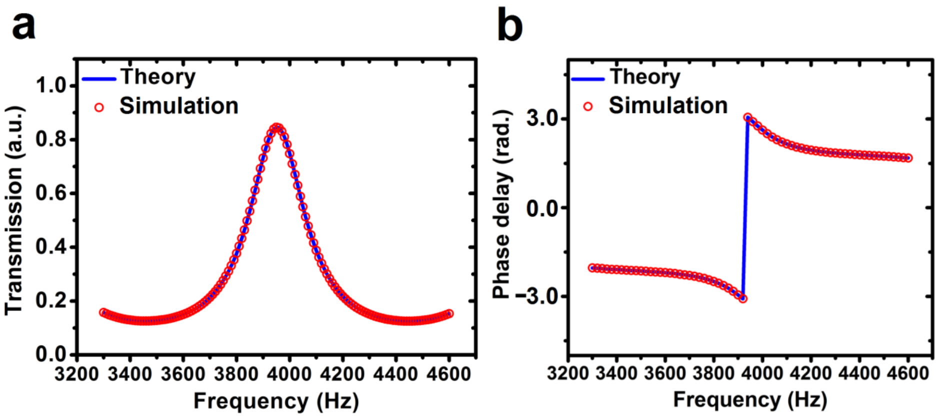

Supplementary Figure 5| Theoretical versus simulation results. **(a)** and **(b)** are the transmission spectra and phase delays, respectively.

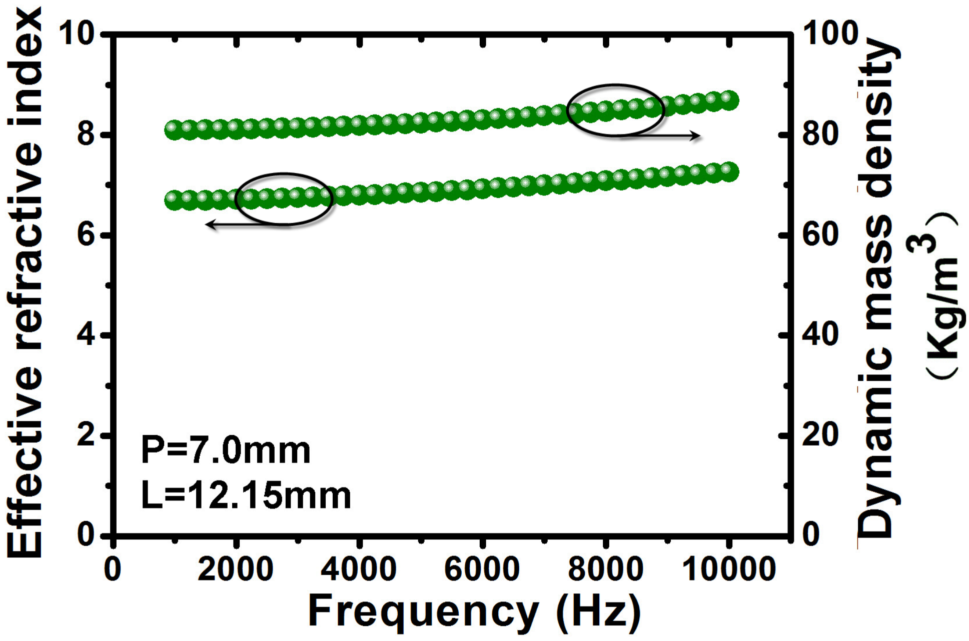

Supplementary Figure 6| The effective refractive index n_{eff} and dynamic mass density ρ_{eff} change with the frequency. We study the sample with L=12.15 mm and P=7.0 mm. When the wavelength is decreasing down to the scale comparable to the sample size (~20 mm), the helical-structured metamaterials can no longer be regarded as a homogenous medium for the incident waves. If we keep on using Eqs. (S18) and (S19) to calculate n_{eff} and ρ_{eff}, we will find out that both n_{eff} and ρ_{eff} increase at high frequency region from 6000 Hz to 10000 Hz and the dispersion-free feature breaks down.

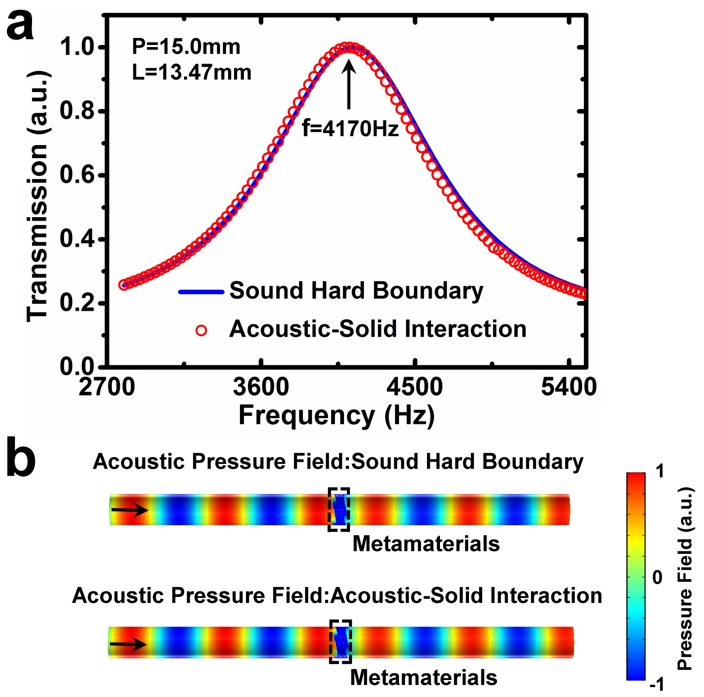

Supplementary Figure 6| The transmission spectra and pressure field distributions. (a) The transmission spectra of metamaterials at sound hard boundary condition and acoustic-solid interaction condition. The material parameters of photopolymer are ρ_p=1190 kg ·m^{-3} for the mass density, E_p=3.2 GPa for the Young's modulus, and σ=0.35 for the Poisson ratio. **(b)** The pressure field distributions at 4170 Hz for those two different boundary conditions. The consistent results suggest that the photopolymer can be regarded as a rigid material for airborne sound.

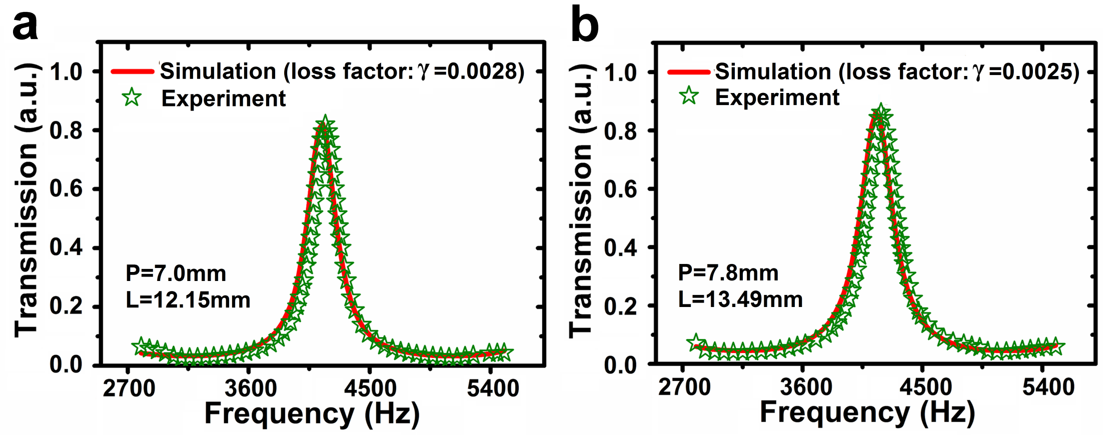

Supplementary Figure 8| The thermo-viscous loss of the helical-structured metamaterials. (a) Numerically simulated and experimentally measured transmission spectra for helical-structured metamaterial unit cell with L=12.15 mm, P=7.0 mm. **(b)** Numerically simulated and experimentally measured transmission spectra for helical-structured metamaterial unit cell with L=13.49 mm, P=7.8 mm. In **(a)**, the loss factor due to thermo-viscous effect γ=0.0028, where the complex sound speed takes the form of $c_0(1+\gamma i)$. In **(b)**, the loss factor due to thermo-viscous effect γ=0.0025.

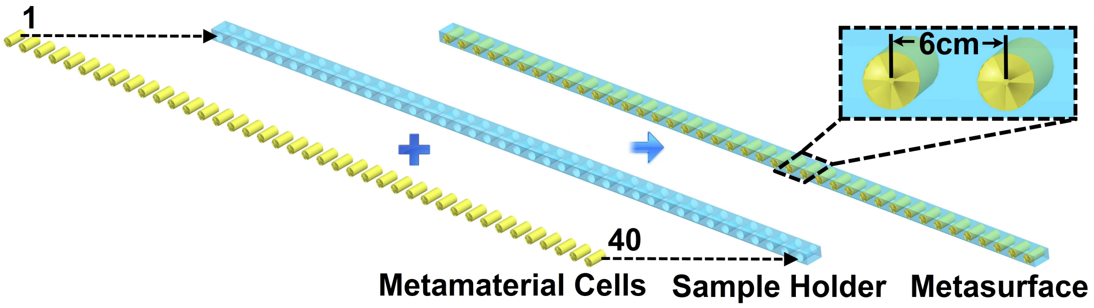

Supplementary Figure 9| The structure of the assembled meta-lens. The helical-structured metamaterial unit cells are numbered and inserted into the sample holder in order. The spacing between neighboring unit cells is 6 cm.

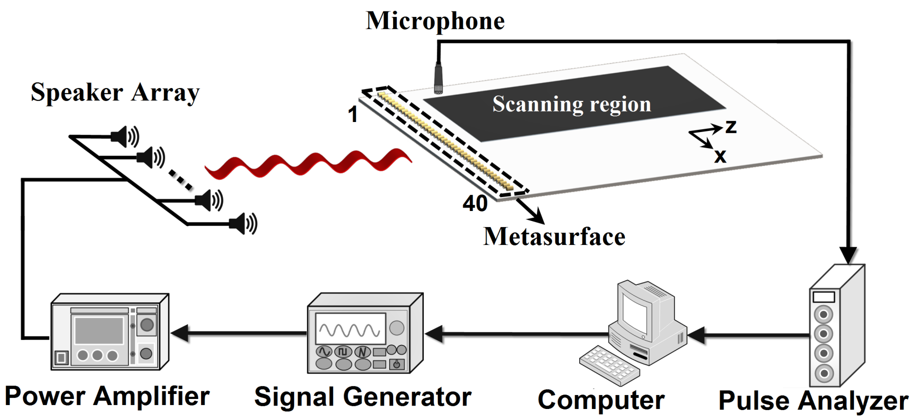

Supplementary Figure 10| The schematic view of the experimental measurement system. Due to the limitations of the translation stage, only the main lobe part of the field is scanned (*see the darken region*).

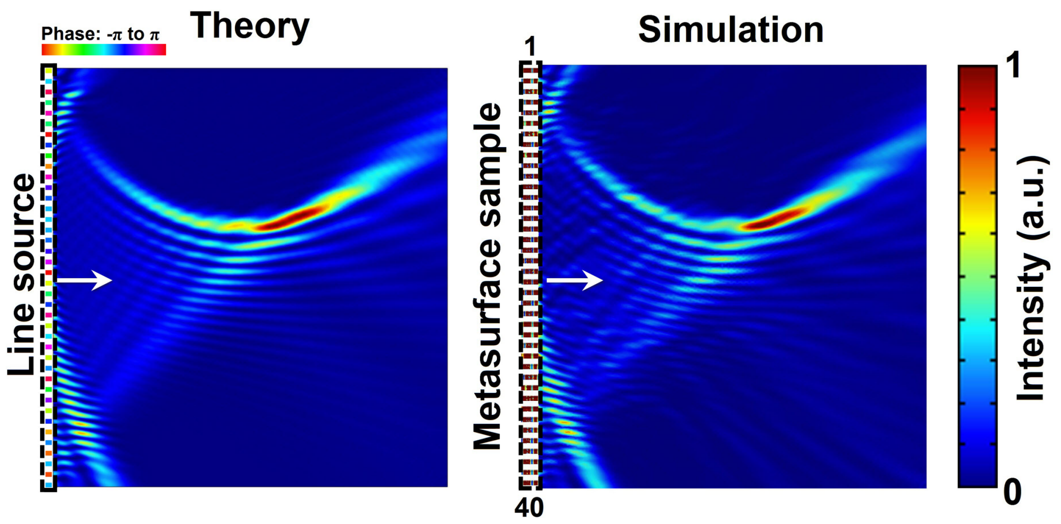

Supplementary Figure 11| The pressure fields of acoustic self-accelerating beams generated directly by a line source with designed phase distributions and formed after the incident plane waves are passing through the acoustic meta-lens (or metasurface). The closely consistent pressure fields for the two different cases demonstrate that the generation of acoustic self-accelerating beam is barely affected by the coupling between adjacent unit cells in the meta-lens.

Supplementary Table 1| Structure parameters and material effective parameters of the unit cells.

Unit cell number	Lead (mm)	Length (mm)	Refractive index	Dynamic mass density (kg·m^{-3})
1	37.80	28.50	1.544	3.466
2	7.00	12.14	6.821	89.024
3	7.00	6.50	6.402	83.708
4	1000.00	41.10	～1	～1.205
5	26.70	38.72	2.078	6.099
6	200.00	23.10	～1	～1.205
7	10.00	9.23	4.518	36.477
8	12.80	21.59	3.844	23.188
9	70.40	31.20	1.099	1.749
10	21.00	18.08	2.353	8.533
11	26.00	37.96	2.120	6.364
12	18.50	29.95	2.786	11.436
13	11.20	19.09	4.344	30.521
14	7.60	13.15	6.288	72.754
15	7.00	12.12	6.821	89.017
16	7.80	13.49	6.125	68.360
17	10.60	18.12	4.572	34.402
18	17.00	27.83	2.994	13.354
19	25.20	38.60	2.175	6.703
20	9.60	8.87	4.694	39.892
21	36.80	27.90	1.569	3.590
22	150.00	23.00	～1	～1.205
23	12.70	21.45	3.872	23.563
24	29.00	41.05	1.955	5.362
25	41.40	30.30	1.464	3.090

26	7.00	12.15	6.821	89.012
27	23.90	35.70	2.261	7.297
28	41.20	30.20	1.467	3.108
29	9.10	15.67	5.282	47.960
30	7.00	6.49	6.401	83.692
31	88.00	32.00	1.019	1.502
32	21.70	34.23	2.445	8.620
33	44.50	31.70	1.403	2.829
34	13.40	22.51	3.689	21.144
35	24.40	20.44	2.093	6.630
36	9.30	16.00	5.173	45.698
37	17.80	15.68	2.695	11.464
38	7.50	12.98	6.371	75.091
39	15.00	13.47	3.121	15.822
40	7.70	13.32	6.206	70.512

Supplementary Note 1. Deduction of the Eqs. (1) and (3) in the manuscript.

The refractive index n of the helical structured metamaterials in Eq. (1) equals to the ratio of the acoustic path in the helical-structured metamaterials over the path in free-space for the same displacement. In Fig. 1a, the sound is propagating helically within the region ($d/2<r<D/2$), where d and D ($d<<D$) are the inner diameter and the outer diameter of the cylindrical metamaterials. Therefore, the equivalent diameter D_e of the helical path of sound is supposed to be smaller than D. Figure 2a shows that the equivalent diameter D_e is related to D through $D_e \approx 0.56D$ after the numerical fitting between Eq. (1) and Eq. (3).

As shown in Supplementary Fig. 3, the lengths of the helical acoustic path in metamaterials l_m and the straight path in free-space l_a are

$$l_m = \sqrt{(\pi D_e)^2 + P^2},$$

$$l_a = P. \tag{1}$$

where D_e is the equivalent diameter of the helical path of sound and P is the thread lead with $P<<D_e$ in the case of high helicity. According to the definition of the refractive index, n can be expressed as follows

$$n = \frac{\sqrt{(\pi D_e)^2 + P^2}}{P} \xrightarrow{P<<\pi D_e} \frac{\pi D_e}{P}. \tag{2}$$

It needs to be mentioned that the term helicity H used in the manuscript can be defined by

$$H = \frac{\sqrt{(\pi D)^2 + P^2}}{P} \xrightarrow{P<<\pi D} \frac{\pi D}{P}. \tag{3}$$

According to Eq. (3), the helicity H is larger when the thread lead P is smaller.

According to the textbook in Supplementary Ref. (2), transmission through a homogeneous medium of length L is

$$T = \frac{4}{4\cos^2\left(k_0 n_1 L\right) + \left(\frac{\rho_1}{\rho_0 n_1} + \frac{\rho_0 n_1}{\rho_1}\right)^2 \sin^2\left(k_0 n_1 L\right)}, \tag{4}$$

where k_0 is the wave vector of sound in air, ρ_0 is the mass density of air, n_1 and ρ_1 are the effective refractive index and dynamic mass density of the homogeneous medium. We will show that n_1 and ρ_1 can be retrieved from the transmission spectrum T. In the transmission spectrum, the Fabry-Pérot resonances will lead to periodic peaks and dips at the conditions of $k_0 n_1 L = N\pi$ and $k_0 n_1 L = (2N-1)\pi/2$, respectively, with N being a positive integer. At the frequency Λ of the first resonant peak in the transmission spectrum, we have

$$k_0 n_1 L = \pi, \tag{5}$$

which can be further formulated into

$$n_1 = \frac{c_0}{2\Lambda L}. \tag{6}$$

At the minimum transmittance (or dips) in the transmission spectrum, we have

$$T_{\min} = \frac{4}{\left(\frac{\rho_1}{\rho_0 n_1} + \frac{\rho_0 n_1}{\rho_1}\right)^2}. \tag{7}$$

From Eq. (7), we finally obtain

$$\rho_1 = \frac{\rho_0 n_1}{\sqrt{T_{\min}}} + \rho_0 n_1 \sqrt{\frac{1}{T_{\min}} - 1}. \tag{8}$$

Supplementary Note 2. Complex transmission coefficients measurement in acoustic impedance tube.

A. *Transfer matrix method.*

In order to obtain the transmission and phase delay of different metamaterial unit cells, we employ the two-load four-microphone method here, which is well known for measuring complex transmission coefficients in an acoustic impedance tube (Supplementary Ref. 3).

The schematic of an acoustic impedance tube is shown in Supplementary Fig. 4. The measured metamaterial unit cell is positioned at the middle of the tube ($0<x<L$). A loudspeaker is mounted at one closed end of the tube, while the other end is open or sealed with a rubber plug (two different loads). It is known that acoustic waves in the impedance tube are simply confined plane waves under the cut-off frequency. Therefore, the complex sound pressures at four different positions (x_1, x_2, x_3, x_4) can be written as

$$\begin{cases} P_1 = Ae^{-ikx_1} + Be^{ikx_1} \\ P_2 = Ae^{-ikx_2} + Be^{ikx_2} \\ P_3 = Ce^{-ikx_3} + De^{ikx_3} \\ P_4 = Ce^{-ikx_4} + De^{ikx_4} \end{cases}, \tag{9}$$

where k represents the wave number in air. A and C are complex amplitudes of the forward propagating plane waves; B and D the complex amplitudes of the backward propagating plane waves. We set the origin of coordinate system at the left facet of the measured unit cell, as shown in Supplementary Fig. 4. The four complex amplitudes of sound pressures can be expressed in terms of the four measured data

$$\begin{cases} A = \dfrac{i(P_1 e^{ikx_2} - P_2 e^{ikx_1})}{2sin(kx_1 - kx_2)} \\ B = \dfrac{i(P_2 e^{-ikx_1} - P_1 e^{-ikx_2})}{2sin(kx_1 - kx_2)} \\ C = \dfrac{i(P_3 e^{ikx_4} - P_4 e^{ikx_3})}{2sin(kx_3 - kx_4)} \\ D = \dfrac{i(P_4 e^{-ikx_3} - P_3 e^{-ikx_4})}{2sin(kx_3 - kx_4)} \end{cases}. \tag{10}$$

The transfer matrix of the metamaterial unit cell is shown as follows

$$\begin{bmatrix} P_0 \\ V_0 \end{bmatrix} = \begin{bmatrix} t_{11} & t_{12} \\ t_{21} & t_{22} \end{bmatrix} \begin{bmatrix} P_L \\ V_L \end{bmatrix}, \tag{11}$$

where L is the overall length of the unit cell, and P_0, P_L, V_0, V_L are the total sound pressures and particle velocities at the two facets of the unit cell, obeying the following relations

$$\begin{cases} P_0 = A + B \\ V_0 = \dfrac{A - B}{\rho_0 c_0} \\ P_L = Ce^{-ikL} + De^{ikL} \\ V_L = \dfrac{Ce^{-ikL} - De^{ikL}}{\rho_0 c_0} \end{cases}, \tag{12}$$

where c_0=343.2 m·s^{-1} and ρ_0=1.2 kg·m^{-3}. In Eq. (11), there are four unknown variables, but only two equations. By employing two different loads at the right end, we can obtain two additional equations for solving the unknown transfer matrix components (t_{ij}). Therefore, the Eq. (11) takes a new form as

$$\begin{bmatrix} P_{0,a} & P_{0,b} \\ V_{0,a} & V_{0,b} \end{bmatrix} = \begin{bmatrix} t_{11} & t_{12} \\ t_{21} & t_{22} \end{bmatrix} \begin{bmatrix} P_{L,a} & P_{L,b} \\ V_{L,a} & V_{L,b} \end{bmatrix}, \tag{13}$$

where subscripts a and b denotes two different load conditions (basically the other end is open or sealed with a rubber plug). From Eq. (13), the transfer matrix

components can be obtained through

$$\begin{bmatrix} t_{11} & t_{12} \\ t_{21} & t_{22} \end{bmatrix} = \frac{1}{P_{L,a}V_{L,b} - P_{L,b}V_{L,a}} \begin{bmatrix} P_{0,a}V_{L,b} - P_{0,b}V_{L,a} & P_{0,b}P_{L,a} - P_{0,a}P_{L,b} \\ V_{0,a}V_{L,b} - V_{0,b}V_{L,a} & P_{L,a}V_{0,b} - P_{L,b}V_{0,a} \end{bmatrix}. \qquad (14)$$

B. *Complex transmission coefficients.*

As shown in Supplementary Fig. 4, the transmission and reflection coefficients are defined by $t = C/A$ and $r = B/A$ for normally incident plane waves. So the Eq. (12) can be reformulated into

$$\begin{cases} P_0 = A(1+r) \\ V_0 = \dfrac{A(1-r)}{\rho_0 c_0} \\ P_L = Ate^{-ikL} \\ V_L = \dfrac{Ate^{-ikL}}{\rho_0 c_0} \end{cases}. \qquad (15)$$

Substituting Eq. (15) into Eq. (11), we obtain the complex transmission coefficients

$$t = \frac{2e^{ikL}}{t_{11} + \dfrac{t_{12}}{\rho_0 c_0} + \rho_0 c_0 t_{21} + t_{22}}. \qquad (16)$$

In order to verify Eq. (16), we conduct numerical calculations based on the finite elements method, where the two loads are perfectly matched layer and hard boundary, respectively. The tested sample is a metamaterial layer with a thickness of 41.1 mm. The sound speed and mass density of the sample are set to be 81.17+0.2*i* m·s^{-1} and 27.49+0.3*i* kg·m^{-3}. The field measurement positions in the left and right parts of the tube are separated by a distance of 7 cm. The theoretically calculated transmission and phase from Eq. (16) are plotted in solid lines in Fig. 5, while the numerical simulation

results are plotted in circles. The good agreement between the theoretical and numerical results validates the feasibility of the two-load four-microphone method for measuring the transmission and phase delay of our designed acoustic metamaterials.

Supplementary Note 3. Deduction of the Eq. (4) in the manuscript based on two-load four-microphone method.

For a homogeneous and isotropic acoustic material with a finite thickness, the 2 ×2 transfer matrix can be given by (Ref. 2)

$$\begin{bmatrix} t_{11} & t_{12} \\ t_{21} & t_{22} \end{bmatrix} = \begin{bmatrix} \cos k_{\mathrm{eff}} L & j\rho_{\mathrm{eff}} c_{\mathrm{eff}} \sin k_{\mathrm{eff}} L \\ j\sin k_{\mathrm{eff}} L / \rho_{\mathrm{eff}} c_{\mathrm{eff}} & \cos k_{\mathrm{eff}} L \end{bmatrix}, \tag{17}$$

where k_{eff} is the complex wave vector of sound in metamaterials, ρ_{eff} and c_{eff} are the effective mass density and speed of sound in metamaterials. Inferred from Eq. (17), the elements of the transfer matrix are related to the material properties. To be specific, the speed of sound in metamaterials can be evaluated as

$$c_{\mathrm{eff}} = \frac{L\omega}{\sin^{-1}(-t_{12}t_{21})}, \tag{18}$$

where ω is the round frequency of the sound. And the mass density of metamaterials can be calculated by

$$\rho_{\mathrm{eff}} = \sqrt{\frac{t_{12}}{t_{21}}} \frac{1}{c_{\mathrm{eff}}}. \tag{19}$$

SUPPLEMENTARY REFERENCES

1. Li, Y., *et al*. Acoustic focusing by coiling up space. *Appl. Phys. Lett.* **101**, 233508 (2012).

2. Allard, J. F. and Atalla, N. *Propagation of Sound in Porous Media: Modelling Sound Absorbing Materials* 2nd edn(John Wiley & Sons Ltd., 2009).

3. Feng, L. P. Modified impedance tube measurements and energy dissipation inside absorptive materials. *Appl. Acoust.* **74**, 1480-1485 (2013).
